# P02.148. Assessing interactions between herbal medicines and drugs: updated review

**DOI:** 10.1186/1472-6882-12-S1-P204

**Published:** 2012-06-12

**Authors:** L Girard, C Necyk, S Jassar, A Filipelli, P Gardiner, H Boon, S Vohra

**Affiliations:** 1 University of Alberta, Edmonton, Canada; 2 Boston University School of Public Health, Boston, USA; 3 Department of Family Medicine, Boston Medical Center, Boston, USA; 4 Leslie Dan Faculty of Pharmacy, University of Toronto, Toronto, Canada

## Purpose

Natural health products (NHPs) are commonly used, both alone and in conjunction with prescription drugs. This concurrent use has been shown to be a concern due to the potential for harmful interactions. A tool to increase awareness of potential interactions between commonly used NHPs and pharmaceuticals was released in 2009. Given the rapid pace of research, it is likely that new interactions have been published since then. The purpose of this project is to review the recent primary and secondary literature for information to update the 2009 tool.

## Methods

Three databases (Medline, Embase, and International Pharmaceutical Academy) were searched for studies conducted from 2007-2010 pertaining to NHP-drug interactions. In addition, National Medicines Comprehensive Database (NMCD) was searched in July 2011. As a cross check tool to verify that no interaction was overlooked, “Herb, nutrient, and drug interactions: clinical implications and therapeutic strategies” (Stargrove et al, 2008) was reviewed to find literature pertaining to NHP-drug interactions. Potentially relevant studies were identified from the primary and secondary literature, and if an interaction was found, the interaction was verified by a second reviewer.

## Results

To date, 1997 studies have been identified from the database search (1910), textbook and NMCD (87) and screened for interactions. Examination of these studies for interactions is ongoing.

## Conclusion

This update is intended to increase the knowledge about NHP-drug interactions as well as to fill in any gaps that may have been overlooked in construction of the original tool. The NHP-drug interaction tool is intended to act as a quick guide for users of NHPs and pharmaceuticals in order to avoid adverse reactions. Future steps in this project will include further updates to the tool as well as creating specific grids for different clinical specialties.

